# Tibetan herbal medicine *Oxytropis falcata* Bunge ameliorates hypoxic pulmonary hypertension in rats via regulation of intestinal microbiota and metabolites

**DOI:** 10.3389/fmicb.2025.1538260

**Published:** 2025-11-28

**Authors:** Yuxin He, Zixu Guo, Hua Xue, Xia Zhu, Tian Luo, Zhenzhong Bai, Lan Ma, Xuefeng Cao

**Affiliations:** 1 Qinghai University Medical College, Xining, China; 2 Research Center for High Altitude Medicine, Qinghai University, Xining, China

**Keywords:** hypoxic pulmonary hypertension, *Oxytropis falcata* Bunge, intestinal microbiota, metabolomics, correlation, mechanism of action

## Abstract

**Background:**

Hypoxic pulmonary hypertension (HPH) is a severe high-altitude disorder with limited therapeutic options. This study investigated the therapeutic mechanisms of *Oxytropis falcata* Bunge (OFB), a traditional Tibetan herbal medicine, in a rat model of HPH, focusing on its effects on endogenous metabolites and gut microbiota.

**Methods:**

HPH was induced in male Sprague–Dawley rats exposed to chronic hypoxia. Animals were randomly assigned to normoxic control, hypoxic model, OFB-treated, or Rhodiola-treated groups. Serum metabolomics (LC-MS) and 16S rRNA sequencing of fecal microbiota were performed. Cardiopulmonary parameters including RVSP and RVHI were assessed, and pulmonary arterial ultrastructure was examined.

**Results:**

OFB significantly attenuated HPH-induced elevations in RVSP and RVHI and mitigated pulmonary arterial remodeling. Metabolomic analysis identified 25 differentially regulated metabolites in HPH, primarily involved in pyrimidine metabolism, which were largely restored by OFB. OFB also reversed HPH-induced gut microbiota dysbiosis, restoring microbial diversity and composition toward normoxic levels. Correlation analysis revealed significant associations between specific bacterial taxa and altered metabolites.

**Conclusions:**

These findings suggest that OFB exerts therapeutic effects against HPH by modulating gut microbiota dysbiosis and restoring metabolic homeostasis, particularly within pyrimidine metabolism. The observed gut–lung axis interactions may underlie these effects, offering novel mechanistic insights and supporting the potential clinical development of OFB as a microbiota-targeted therapy for HPH.

## Introduction

Pulmonary arterial hypertension (PAH) is frequently referred to as the “cancer” of the cardiovascular system, with hypoxic pulmonary hypertension (HPH) being particularly prevalent among high-altitude populations. Chronic hypoxia has been demonstrated to induce persistent pulmonary vasoconstriction and pulmonary artery remodeling. This, in turn, has been shown to lead to increased pulmonary circulation resistance and right heart failure (Wan et al., 2024). *Oxytropis falcata* Bunge, a traditional Tibetan medicine known as “Edaxia,” has been widely used for its anti-inflammatory and hemostatic properties. Recent studies have indicated a close relationship between the gut microbiota and metabolites in patients with pulmonary arterial hypertension (PAH) (Gao et al., 2023; Ailizire et al., 2023; Wang et al., 2023). However, the specific mechanisms by which OFB regulates gut microbiota and metabolites in HPH remain to be elucidated. This study employs an innovative approach to investigate the therapeutic effects of OFB on HPH. It does this by analyzing changes in serum metabolites and gut microbiota composition, thus providing novel insights into the pathophysiology of HPH. Moreover, recent research has indicated that the gut-brain-lung axis may potentially have a significant role in the development of HPH (Kim, 2025). For instance, the identification of a postprandial neuroimmune axis by Chen et al. provides a link between the gastrointestinal tract and lung function via brain-coordinated sensory and motor circuits. This may offer a potential connection to the gut-lung axis in HPH (Kim, 2025). Furthermore, the role of specific gut microbiota taxa and their corresponding metabolites in modulating the overall metabolic profile in HPH warrants further investigation. The objective of this study is to address these knowledge gaps and to provide a comprehensive understanding of the therapeutic potential of *Oxytropis falcata* Bunge in HPH.

## Materials and methods

### Instruments and reagents

The following reagents and instrumentation were utilized in the study: LC–MS grade methanol, acetonitrile, ammonia, and isopropanol (CNW Technologies); Phusion Hot start flex 2X Master Mix (Shanghai Yitao Biological Instrument Co., LTD); DL2000 DNA Marker (Takara); Gene color (Beijing Jinboyi); Biowest Agarose G-10 (BIOWEST); AMPure XT beads (Beckman); NovaSeq 6000 sequencer (Illumina); centrifuge (Thermo Fisher Scientific); ultrasonic instrument (Shenzhen Redbond Electronics Co., Ltd.); homogenizer (Shanghai Jingxin Technology Co., Ltd.); freeze dryer (Sihuan Furuike Technology Development Co., Ltd.); Qubit (Invitrogen Q33226); Vanquish (Thermo Fisher Scientific).

### Preparation of alcoholic extracts from the traditional Chinese medicine *Oxytropis falcata* Bunge

The whole grass powder of *Oxytropis falcata* Bunge (3,060 g, batch number T630602174) was obtained from Qinghai Tibetan Hospital and authenticated by Duojie Cairang, the chief pharmacist. The powder was reflux-extracted in a 10 L extraction device with 95% ethanol at a liquid-to-solid ratio of 10:1. The extraction was performed at 75 °C for 3 h, repeated three times. The combined extracts were concentrated using a rotary evaporator, centrifuged at 3,000 rpm for 5 min, and the supernatant was dried at 80 °C for 4 h. The final extract was freeze-dried into a powder and stored at −20 °C.

### Preparation of *Rhodiola* alcohol extract

The *Rhodiola* decoction pieces were ground into powder, loaded into a 10 L extractor already containing *Rhodiola rosea*, and reflux-extracted three times (75 °C, 60 min each) with ethanol–water 1:100. After extraction the pooled mixture was mixed, filtered, centrifuged (3,000 rpm, 5 min) and distilled; the filtrate was transferred to a glass petri dish and dried at 80 °C for 4 h to give an alcohol extract that was kept at −20 °C, then freeze-dried in a 50 mL centrifuge tube to yield a dry powder.

### Animals and grouping

Forty male Sprague–Dawley rats (weighing 130–150 g) were procured from Beijing Weitong Lihua Company (license number: SCXK (Beijing) 2021-0006). The rats were acclimatized for a period of 7 days prior to being divided into five groups: the normoxic control group (nor), the hypoxic simulation group (hyp), the hypoxic OFB group (hypofb), and the hypoxic *Rhodiola* group (hyprho). The normoxic control group was housed in the SPF-grade animal laboratory of Qinghai University, with a temperature of (22 ± 2)°C, humidity of (50 ± 10)%, and a 12 h light–dark cycle, with free access to food. The remaining three groups were accommodated in the low-pressure oxygen chamber (DYC3000, Guizhou Fenglei Aviation Ordnance Co., Ltd.) at Qinghai University, with a temperature of (24 ± 2)°C, humidity of (55 ± 10)%, and a 12 h light–dark cycle, with free access to food. All experimental procedures were approved by the Ethics Committee of Qinghai University Medical School (approval number: SL202401-51).

### Plasma sample processing and metabolite extraction

Blood samples were collected from the aorta under urethane anesthesia and subsequently subjected to centrifugation at 3000 g for 10 min at 4 °C. The serum was then divided into 200 μL aliquots and stored at −80 °C. For the extraction of metabolites, 100 μL of plasma was mixed with 400 μL of an extraction solution (methanol: acetonitrile = 1:1, V/V) containing an internal standard. The mixture was subjected to a vortex for a period of 30 s, followed by an incubation in an ice bath for a duration of 10 min. Subsequently, the mixture was subjected to a centrifugation process at a speed of 12,000 rpm for a duration of 15 min at a temperature of 4 °C. The sample was transferred to a vial for the purpose of liquid chromatography-mass spectrometry (LC–MS) analysis.

### Liquid chromatography-mass spectrometry conditions

The separation of polar metabolites was achieved through the utilization of a Waters ACQUITY UPLC BEH Amide column (2.1 mm × 50 mm, 1.7 μm), employing a Vanquish ultra-high-performance liquid chromatograph. The mobile phase A solution comprised 25 mmol/L ammonium acetate and 25 mmol/L ammonia, while the mobile phase B solution was acetonitrile. The temperature of the sample tray was maintained at 4 °C, and the injection volume was 2 μL. The acquisition of mass spectrometry data was conducted using an Orbitrap Exploris 120 mass spectrometer, which was controlled by Thermo’s Xcalibur software (version 4.4).

### Processing of intestinal contents samples and 16S rRNA sequencing

Intestinal contents were collected from the cecum of rats that had been anesthetized and stored at −80 °C. The DNA was extracted using the CTAB method, and its purity and concentration were assessed by agarose gel electrophoresis and UV spectrophotometry, respectively. The 16S rRNA V3-V4 region was subjected to PCR amplification using specific primers, and the resulting PCR products were purified and sequenced on a NovaSeq 6000 platform (Illumina) with a 2 × 250 bp paired-end configuration.

### Data processing and statistical analysis

The raw LC–MS data were converted to mzXML format using the ProteoWizard program and processed using the R package. A total of 30,346 peaks were extracted from 5 QC samples and 40 experimental samples. Following the implementation of data preprocessing procedures, incorporating the removal of outliers, the imputation of missing values, and normalization, a total of 18,871 peaks were retained for subsequent analysis. The 16S rRNA sequencing data were processed using DADA2 for denoising and ASV identification. Alpha and beta diversity analyses were performed using various indices and distance metrics. The identification of species was conducted using the SILVA database. Statistical significance was determined using Fisher’s exact test, Mann–Whitney U test, or Kruskal-Wallis test, with *p* < 0.05 considered significant.

## Results

### Right ventricular systolic blood pressure (RVSP) and right cardiac hypertrophy index (RVHI)

**Figure 1:**
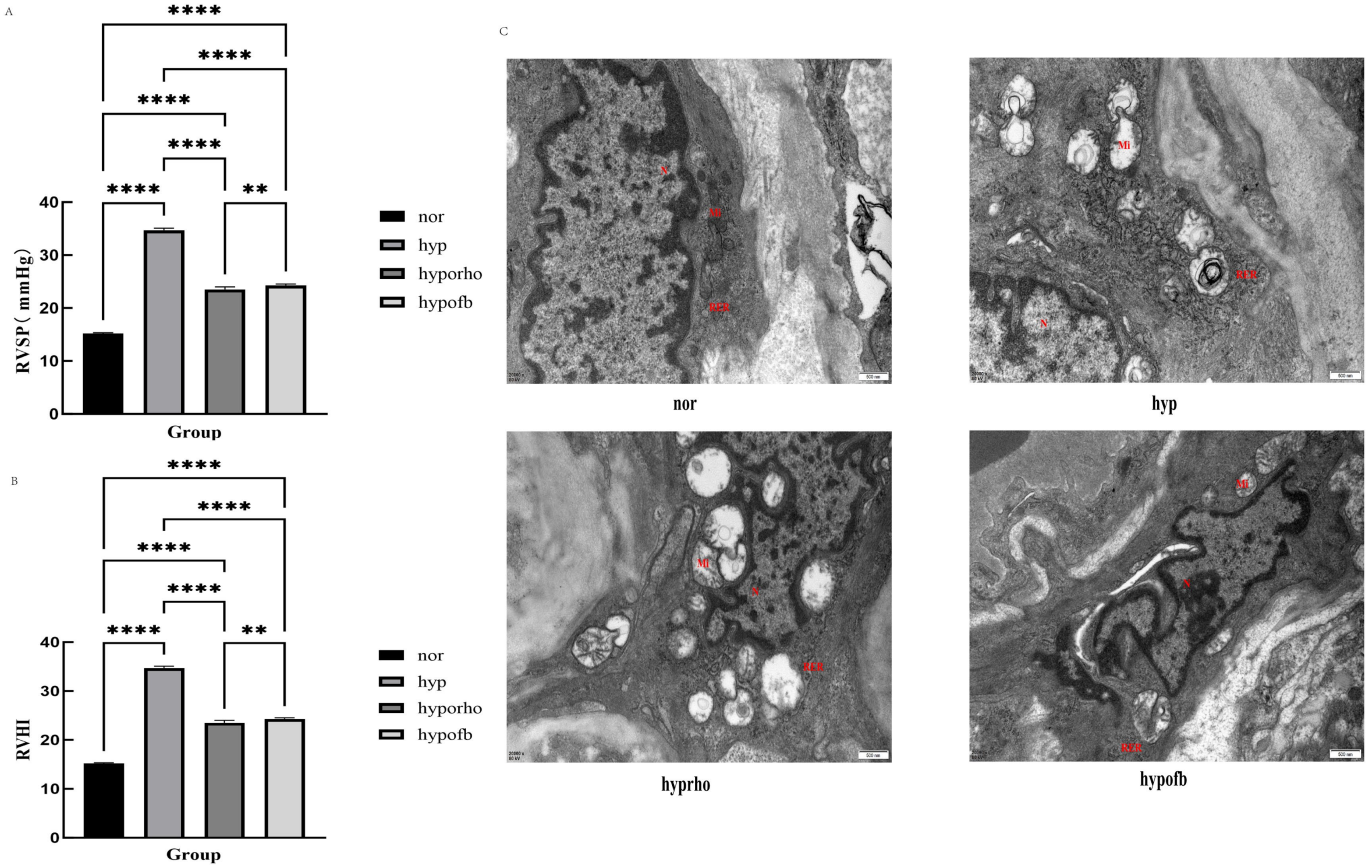
Effects of *Oxytropis falcata* Bunge on cardiopulmonary pathological indicators in rats with hypoxic pulmonary hypertension (HPH). **(A)** Right ventricular systolic blood pressure (RVSP) in rats; **(B)** right ventricular hypertrophy index (RVHI) in rats; **(C)** transmission electron microscopy results of pulmonary artery smooth muscle cells.

In comparison with the normoxic control group, the hypoxic simulation group demonstrated a marked increase in RVSP (*p* < 0.001) and RVHI (*p* < 0.001). The findings revealed that both OFB and *Rhodiola* treatments significantly reduced RVSP and RVHI in HPH rats (*p* < 0.001 for OFB, *p* < 0.01 for *Rhodiola*), thereby indicating their protective effects against HPH-induced right heart damage (Figure 1).

### Transmission electron microscopy of rat lung smooth muscle cells

Transmission electron microscopy revealed significant remodeling of pulmonary artery smooth muscle cells in the hypoxic simulation group, characterized by mitochondrial proliferation, swelling, and rough endoplasmic reticulum expansion. The administration of OFB treatment resulted in a significant alleviation of these pathological changes, with a more pronounced effect than *Rhodiola* (Figure 1).

### Serum differential metabolite sceening

**Figure 2:**
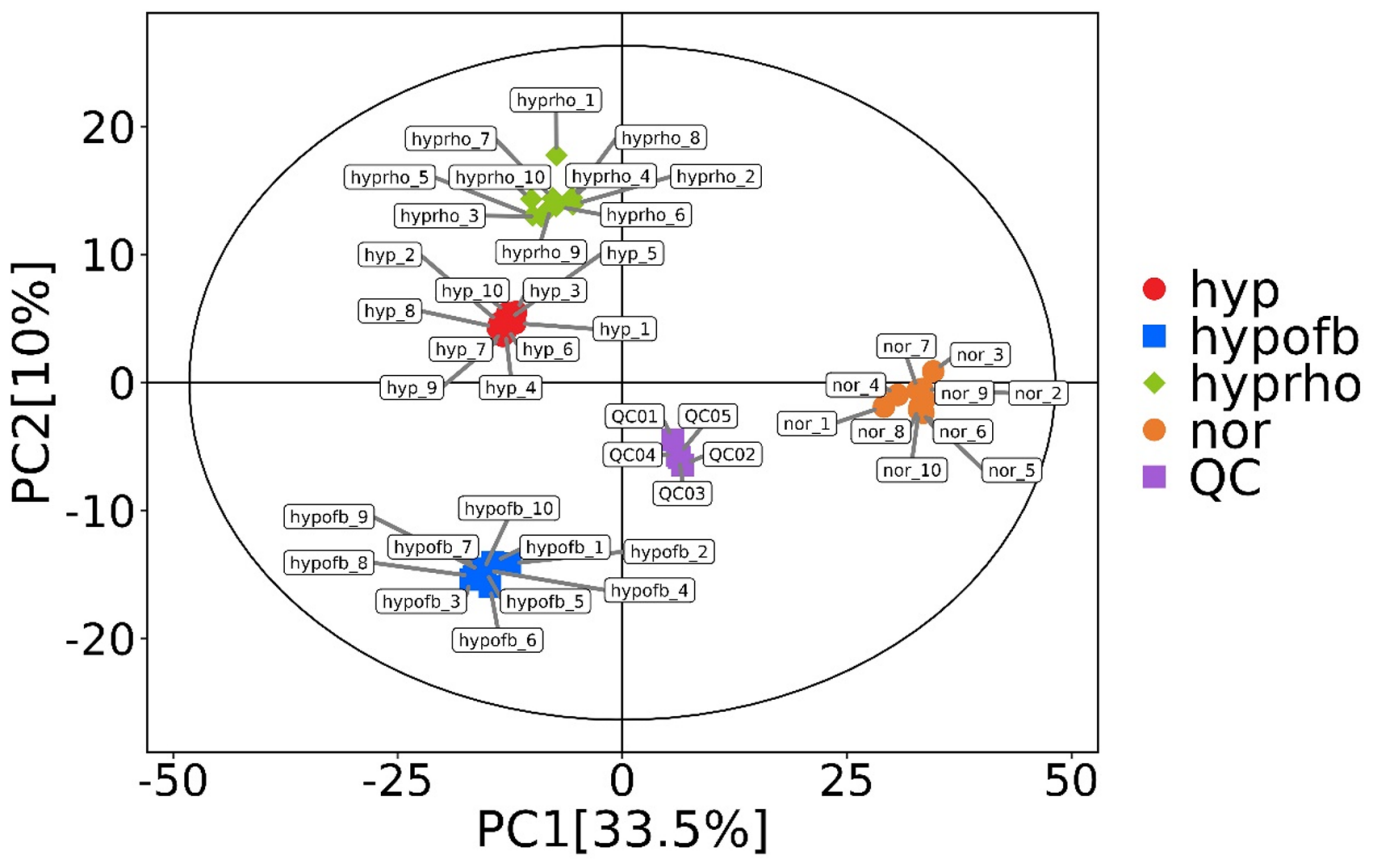
Principal component analysis (PCA) plot.

**Figure 3:**
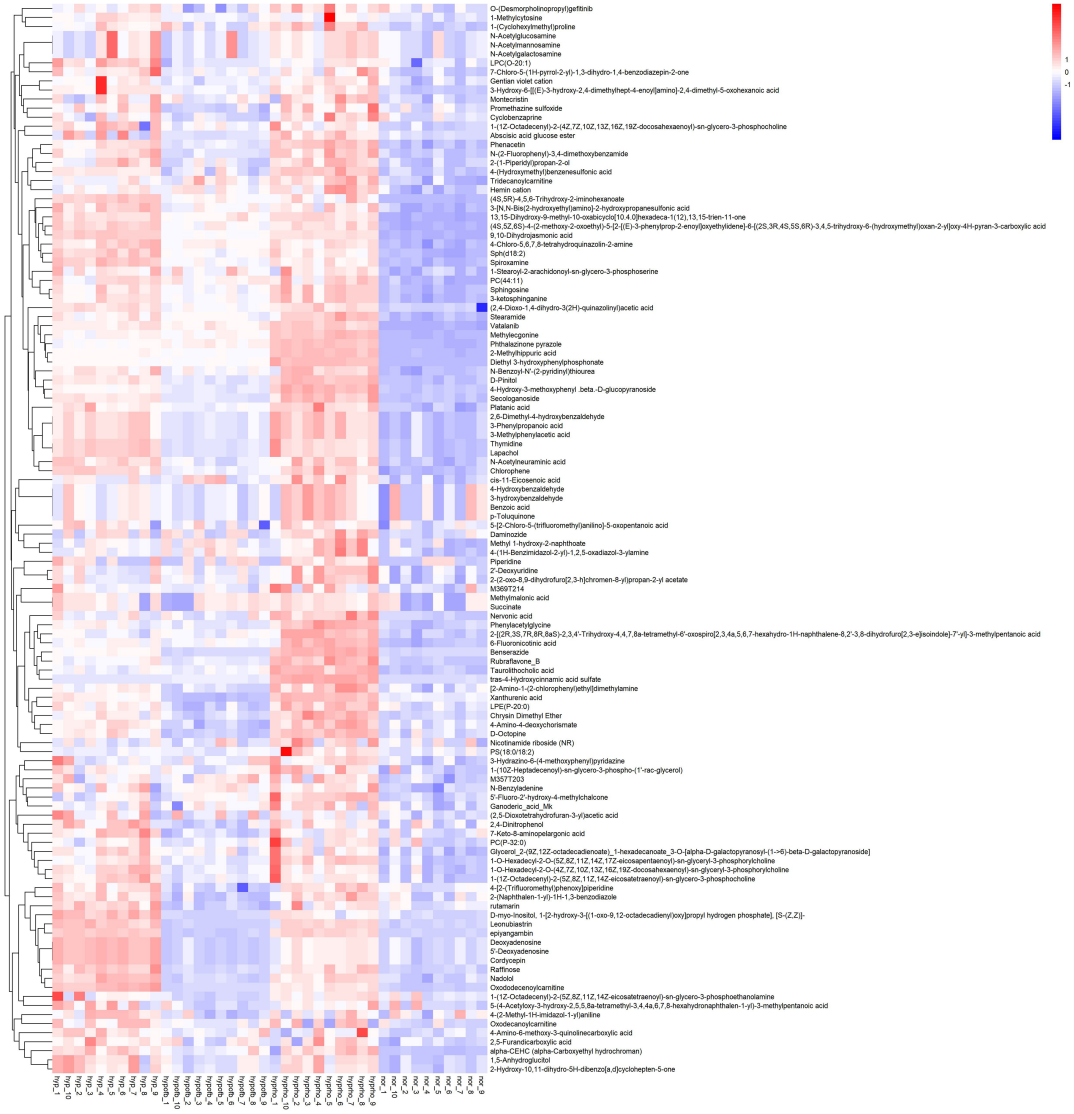
Volcano plot of the top 25 differential metabolites.

**Figure 4:**
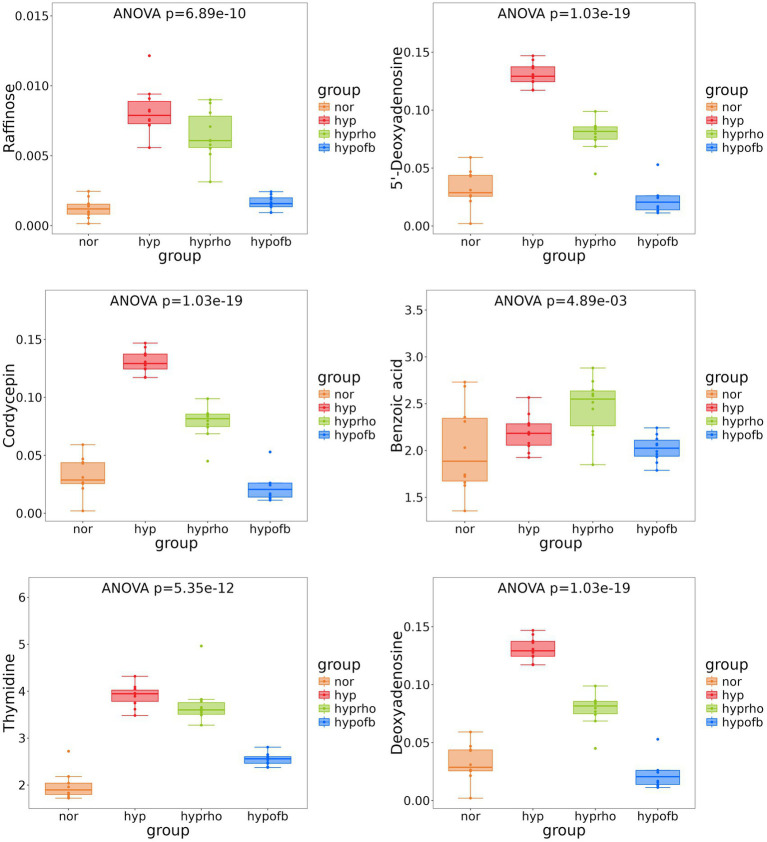
Box plots of six differential metabolites.

**Figure 5:**
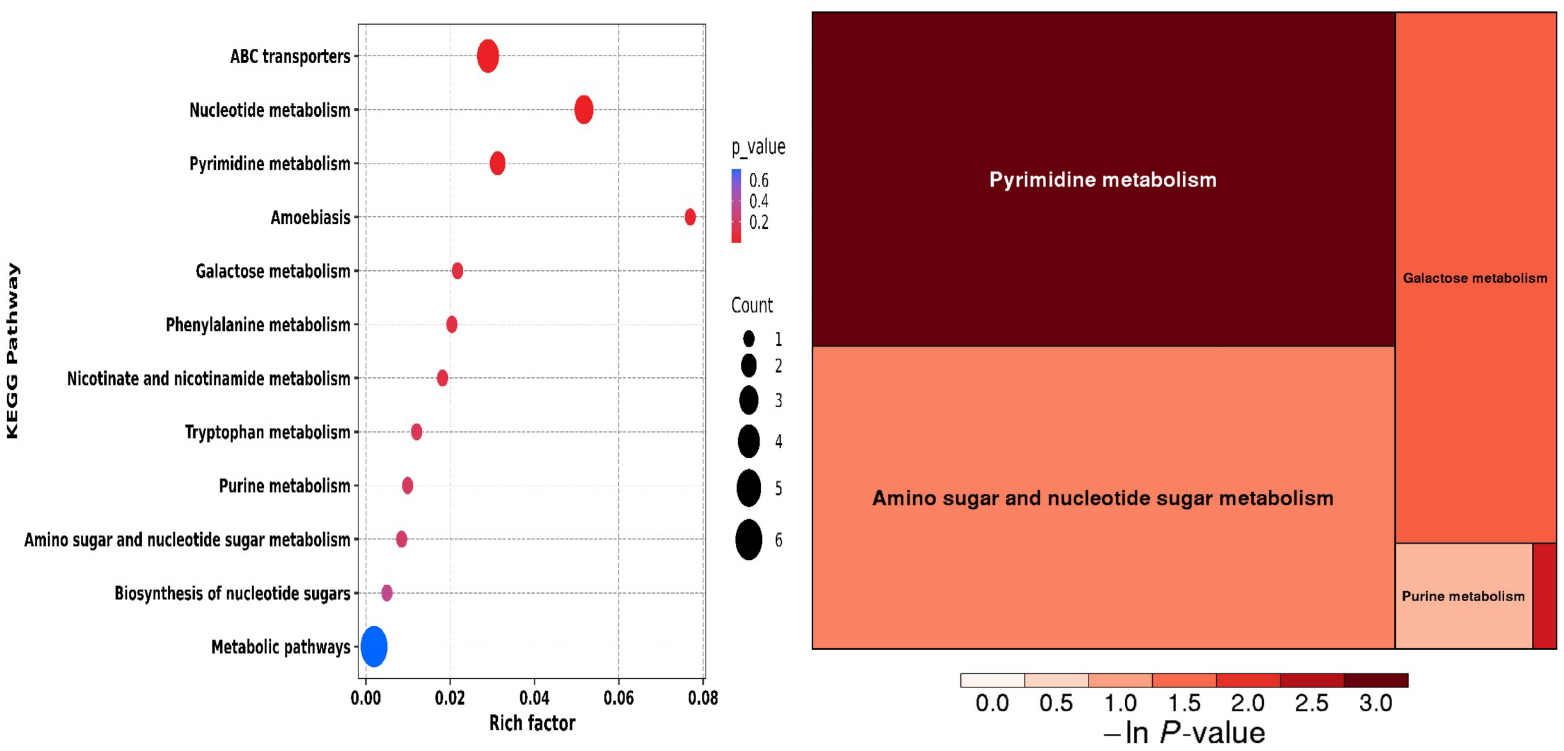
Differential metabolite enrichment pathways.

Principal component analysis (PCA) and clustering heatmap analysis revealed significant disparities in endogenous metabolites between the hyp and the nor groups. A total of 25 differential metabolites were identified, primarily involved in the pyrimidine metabolism pathway. Treatment with OFB resulted in the restoration of metabolite levels to those observed in the normoxic control group (see Table 1; Figures 2–5).

**Table 1:** The top 25 differential metabolites.

ID	MS2 name	MS2 score	Level	mz	rt	Type	MEAN hyp	MEAN hypofb	MEAN hyprho	MEAN nor	ANOVA *p*-value	*Q*-value
32	Raffinose	3.97	Level 1	527.1583	252.9	POS	0.008218351	0.001635189	0.00646667	0.001236463	6.89406E-10	3.34E-09
171	5’-Deoxyadenosine	3.94	Level 1	252.109	55.4	POS	0.130666656	0.022517605	0.078347052	0.032559336	1.03147E-19	3.66E-18
221	Cordycepin	3.92	Level 1	252.109	55.4	POS	0.130666656	0.022517605	0.078347052	0.032559336	1.03147E-19	3.66E-18
242	Benzoic acid	3.92	Level 1	121.0293	39.9	NEG	2.191028666	2.021285808	2.463666409	2.021923684	0.004894253	0.008377
243	Thymidine	3.92	Level 1	241.0827	35.9	NEG	3.90441324	2.547981721	3.725624402	1.986362147	5.34753E-12	3.75E-11
250	Deoxyadenosine	3.91	Level 1	252.109	55.4	POS	0.130666656	0.022517605	0.078347052	0.032559336	1.03147E-19	3.66E-18
259	N-Acetylneuraminic acid	3.91	Level 1	290.0879	202.2	NEG	0.029749746	0.025554609	0.029824032	0.022719959	3.40952E-13	2.98E-12
304	Taurolithocholic acid	3.9	Level 1	482.2939	26.3	NEG	0.020541886	0.020094269	0.049405946	0.003095705	3.45325E-20	1.36E-18
334	2’-Deoxyuridine	3.89	Level 1	227.067	41.6	NEG	1.556616267	1.507789339	1.925120462	1.316344412	1.80474E-05	4.44E-05
352	Phenylacetylglycine	3.88	Level 1	194.0811	104.8	POS	0.193322056	0.159471961	0.279869696	0.123887345	1.15244E-14	1.4E-13
360	N-Acetylgalactosamine	3.87	Level 1	204.0866	146.2	POS	0.402183004	0.37066837	0.410051429	0.360002197	0.002681286	0.004761
392	Nicotinamide riboside (NR)	3.85	Level 1	255.0978	226.4	POS	0.279892593	0.279475395	0.374718317	0.277570351	0.006158539	0.010355
408	Xanthurenic acid	3.84	Level 1	204.03	104.4	NEG	0.079122611	0.043985958	0.102317366	0.064310537	5.63329E-26	7.65E-24
413	N-Acetylglucosamine	3.83	Level 1	204.0866	146.2	POS	0.402183004	0.37066837	0.410051429	0.360002197	0.002681286	0.004761
565	Lapachol	3.53	Level 1	241.0827	35.9	NEG	3.90441324	2.547981721	3.725624402	1.986362147	5.34753E-12	3.75E-11
586	Phenacetin	2.75	Level 2	180.1019	141.5	POS	0.461074176	0.383078371	0.512193435	0.319338898	1.14796E-18	3.3E-17
646	Nadolol	2.74	Level 2	310.2012	130.2	POS	1.936454386	1.38384633	1.790481805	1.366263405	1.37077E-17	3.11E-16
863	Benserazide	2.71	Level 2	258.1083	55.9	POS	0.180787371	0.051587566	0.373097467	0.050143977	3.3767E-17	7.05E-16
1,117	2,4-Dinitrophenol	2.6	Level 2	183.0063	32.4	NEG	0.097521645	0.079663164	0.093162017	0.083317728	0.000105028	0.000232
1,315	Chlorophene	2.5	Level 2	217.0475	167.3	NEG	0.713553081	0.608223836	0.708274083	0.505426854	2.32234E-11	1.45E-10
1,341	Tras-4-Hydroxycinnamic acid sulfate	2.5	Level 2	242.9966	109.2	NEG	0.913208656	1.067196329	3.918269247	0.632303958	9.88622E-18	2.32E-16
1,375	2,5-Furandicarboxylic acid	2.49	Level 2	155.0016	358.2	NEG	0.54303988	0.498636417	0.534486538	0.451627382	0.002326751	0.004176
1,657	Rubraflavone_B	2.27	Level 2	475.247	179.1	POS	0.143104072	0.124742857	0.213919486	0.095587319	3.21286E-10	1.64E-09
2,024	(2,5-Dioxotetrahydrofuran-3-yl)acetic acid	2	Level 2	157.0115	134.1	NEG	0.055752903	0.04840304	0.05086359	0.039586779	0.016237543	0.025452
2,158	Abscisic acid glucose ester	1.34	Level 3.1	447.163	62	NEG	0.022306658	0.010674762	0.024992297	0.00928055	6.83842E-06	1.79E-05

ID: the unique data number for qualitative analysis in this experiment; MS2 name: the name of the substance obtained from MS2 qualitative matching analysis; MS2 score: the mean value of relative quantification in one experimental group of the group comparison; level: the qualitative level of the substance; mz: the mass-to-charge ratio of the characteristic ion of the substance; rt: the chromatographic retention time of the substance; type: the acquisition mode MEAN (group): the mean value of relative quantification in one experimental group of the group comparison; ANOVA *p*-value: the *p*-value obtained from the analysis of variance of the substance in the group comparison; *Q*-value: the result of the multiple hypothesis testing correction of the hypothesis testing statistic (*p*-value).

### Gut microbiota composition

**Figure 6:**
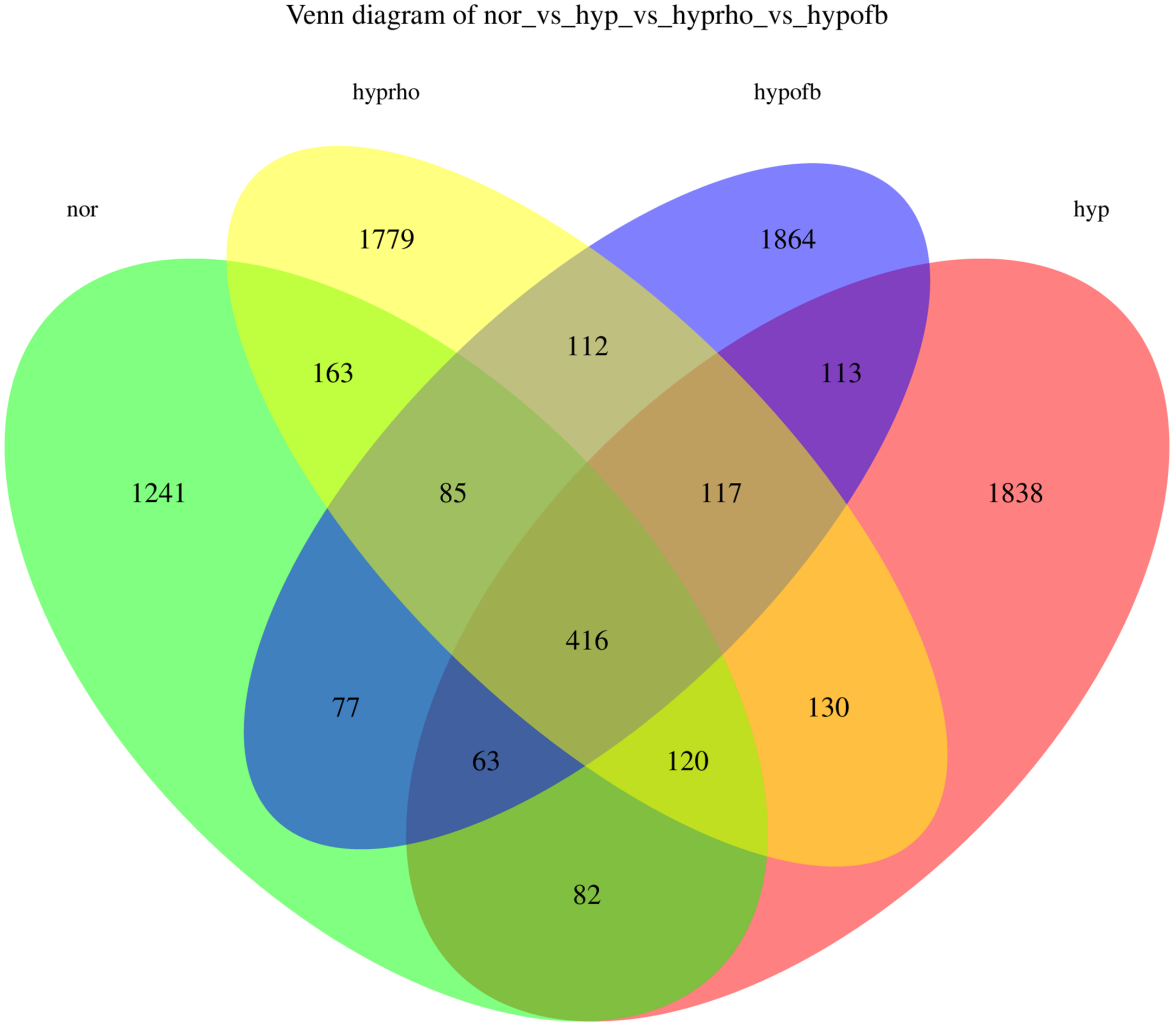
Venn diagram.

**Figure 7:**
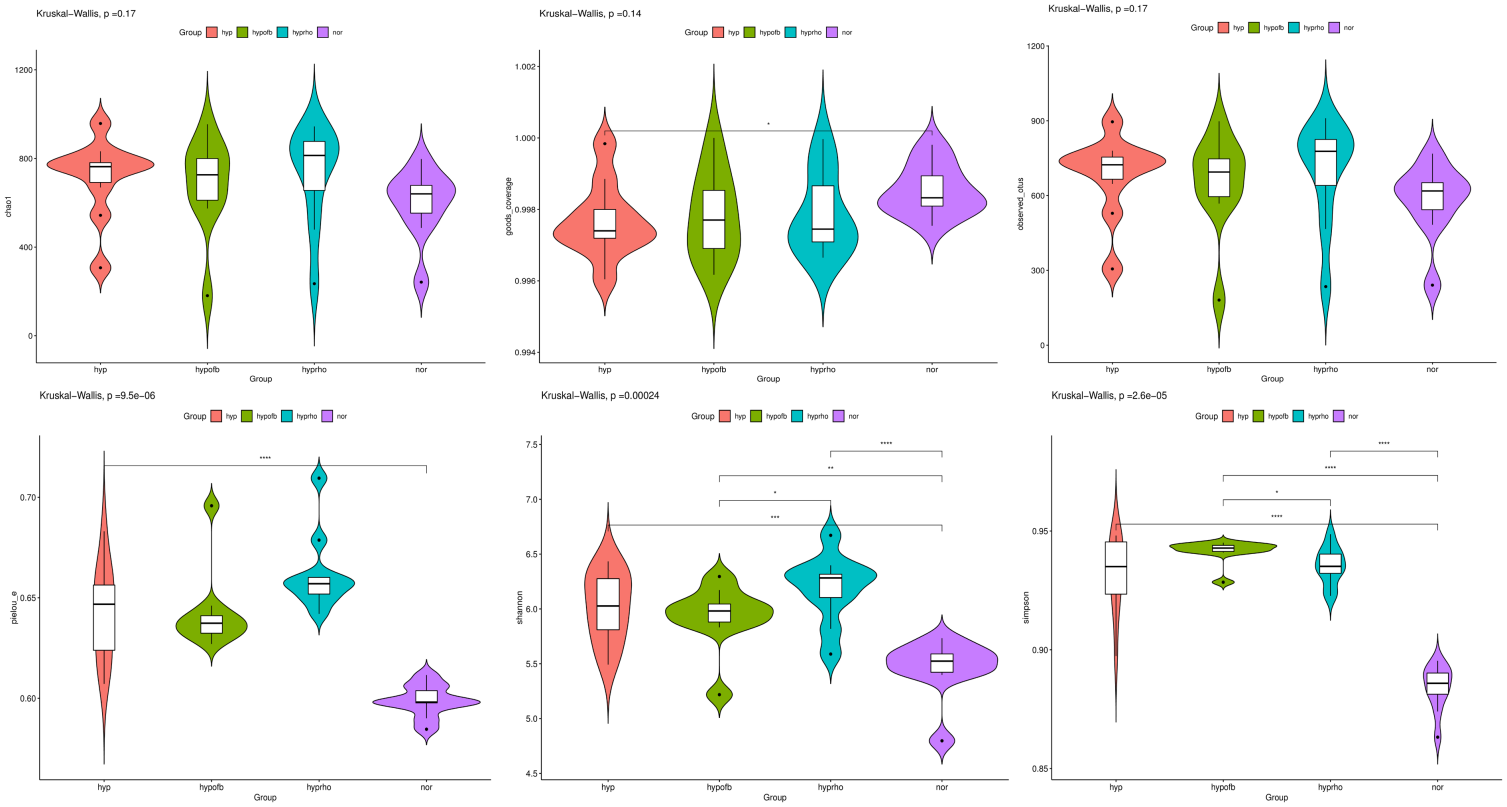
Distribution of alpha diversity indices (Chao1 Index, Goods Coverage, Observed Species Index, Simpson Diversity Index, and Shannon Diversity Index) among different treatment groups.

The results from the Venn diagram indicate significant differences in microbial community composition among the various treatment groups, with certain species being more abundant or unique to specific treatment groups. The violin plots for the Shannon and Simpson diversity indices demonstrate that the hypoxic group exhibits significantly higher microbial diversity (richness and evenness) compared to other groups (*p* < 0.05). In contrast, the violin plots for the Goods Coverage, Chao1 index, and Observed Species index demonstrate no significant differences in species coverage, total species count, and the number of observed species among the different groups (*p* > 0.05). Combining the Venn diagram analysis with previous findings, it is hypothesized that OFB treatment may contribute to restoring the diversity of the gut microbiota, bringing it closer to that of the normoxic control group (see Figures 6, 7).

### Beta diversity and species analysis

**Figure 8:**
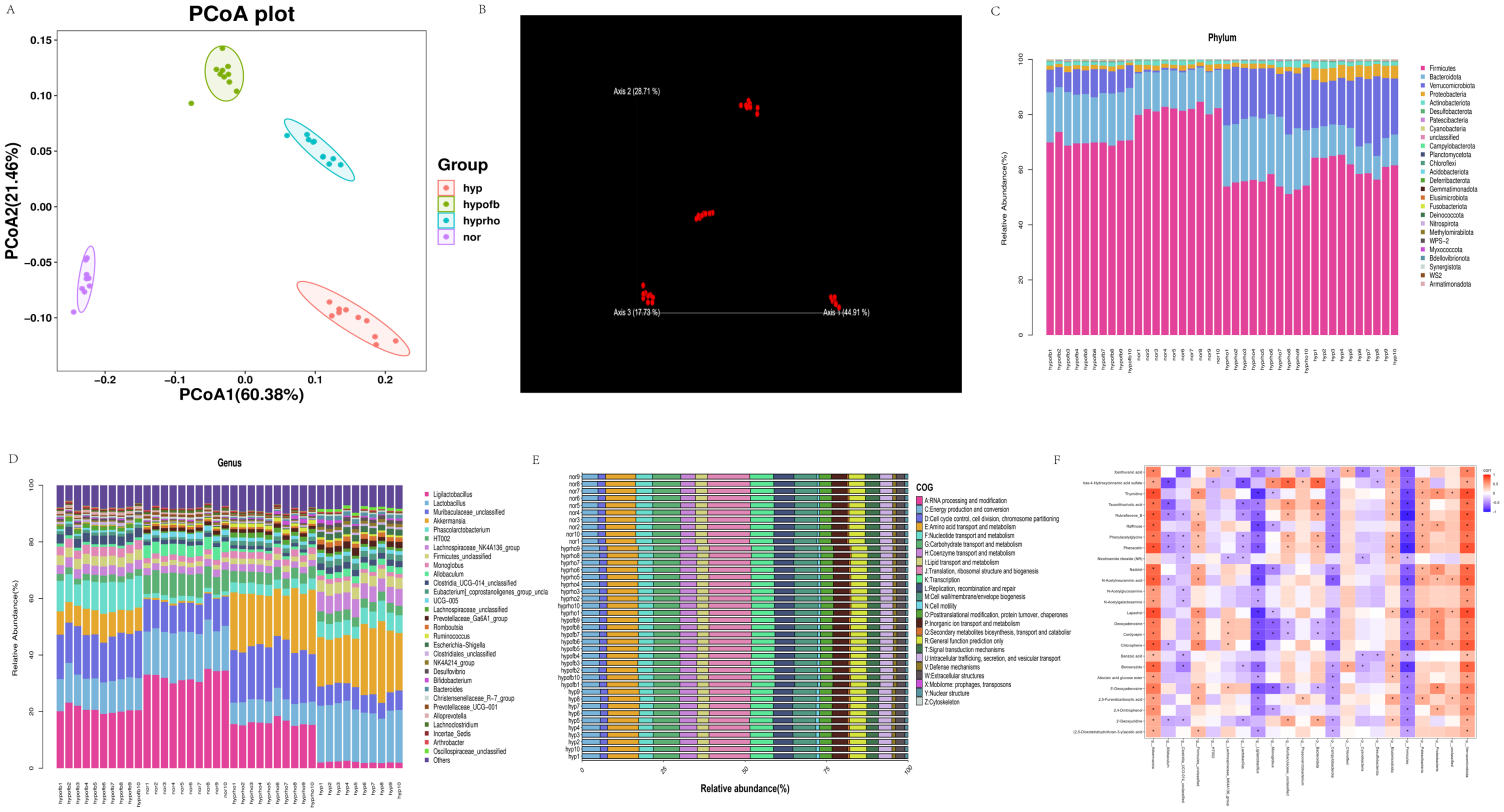
**(A)** β diversity analysis of gut microbiota using Principal Coordinate Analysis (PCoA); **(B)** β diversity analysis of gut microbiota using Non-metric Multidimensional Scaling (NMDS); **(C)** stratified map of the top 30 intestinal flora richness of phylum and genus; **(D)** stratified map of the top 30 intestinal flora richness of phylum and genus; **(E)** COG functional classification of gut microbiota; **(F)** heat map of correlation analysis between differential metabolites and intestinal flora.

PCA and PCoA analysis revealed substantial disparities in the composition of gut microbiota between the normoxic and hypoxic groups. The hypofb group demonstrated a microbiota composition that was more akin to that of the normoxic group, thus suggesting that OFB exerts a therapeutic effect on gut microbiota dysbiosis in HPH rats (Figure 8).

### The relative abundance and functional analysis of gut microbiota

The application of the COG database to the functional classification of gut microbiota has revealed that the microbiota in HPH rats is involved in a variety of biological processes, including energy metabolism, RNA processing, and cell cycle regulation. The administration of OFB treatment resulted in a substantial alteration of the functional profile of the gut microbiota, leading to its restoration to a state resembling normoxia (Figure 8).

### Correlation analysis between gut microbiota and metabolites

Spearman correlation analysis revealed significant correlations between specific gut microbiota taxa and serum metabolites. For instance, *N-acetylglucosamine* demonstrated a positive correlation with *Verrucomicrobiota* and a negative correlation with *Firmicutes*. These correlations highlight the interplay between gut microbiota and host metabolites in HPH (Figure 8).

## Discussion

This study explores the therapeutic effects of *Oxytropis falcata* Bunge on hypoxic pulmonary hypertension (HPH) by analyzing changes in serum metabolites and gut microbiota. The findings demonstrate that OFB can regulate gut microbiota dysbiosis and restore metabolic homeostasis in HPH rats. As demonstrated in preceding studies, an imbalance in the composition of the gut microbiota has been demonstrated to be associated with the progression of pulmonary hypertension (Callejo et al., 2018; Baix et al., 2022; Everard et al., 2013). The present study revealed that the abundance of *Firmicutes* and *Lactobacillus*, two dominant phyla in the gut microbiota, was significantly reduced in the hypoxic group. However, OFB treatment was found to restore their abundance to normal levels. In this study, researchers observed that treatment with OFB significantly restored the diversity of the gut microbiota and the levels of metabolites in a rat model of HPH. This finding is in accordance with the results obtained by Rastogi et al., who proposed that gut microbes ferment dietary fibers to produce metabolites, particularly short-chain fatty acids (SCFAs) (Rastogi et al., 2022). These SCFAs have been shown to migrate directly to lung tissues through the bloodstream, modulating pulmonary immune responses, or promoting the differentiation and activation of immune cells to produce cytokines and IgA. In this experiment, the administration of OFB treatment resulted in a significant reduction in HPH-induced damage, which may be partly attributable to the effects of SCFAs. It is evident that SCFAs exert their influence not only within the gastrointestinal tract but also potentially on the pulmonary immune environment via the gut-lung axis (Rastogi et al., 2022; Dumas et al., 2018; Anand and Mande, 2018; Price and O'Toole, 2021; Ashique et al., 2022). In the lungs, IgA has been shown to promote the clearance of pathogens, regulatory T cells (Tregs) have been demonstrated to reduce pulmonary inflammation and injury, and certain cytokines (such as TNF-*α*, IL-4, etc.) have been observed to alter the immune environment, thereby affecting lung health. These results suggest that OFB may improve the imbalance of gut microbiota and metabolite levels in HPH rats by regulating the gut microbiota and its metabolites, thereby positively impacting lung health through the gut-lung axis. It can be concluded from these findings that OFB may exert its therapeutic effects by modulating the gut microbiota (Luo et al., 2023; Sanada et al., 2020; Liao et al., 2023; Khanna et al., 2021; Luo et al., 2024).

The association between lung injury and neurocognitive dysfunction is a subject that is currently receiving increased attention from the scientific community. The “triple-hit” hypothesis is a theoretical model that has been postulated as a potential underlying mechanism (Cheng et al., 2024). This hypothesis elucidates a cascade of events triggered by lung injury, including immune dysregulation, inflammation, and microbiota changes, which collectively activate the “lung-gut axis,” leading to the onset or exacerbation of cognitive impairments. The “gut-lung axis” and “gut-brain axis” are used to denote the complex interactions between the gut microbiota and the lungs, as well as between the gut microbiota and the brain, respectively. Dysbiosis of the gut microbiota has been demonstrated to facilitate the migration of pathogenic bacteria to the lungs and modulate lung immune responses, potentially contributing to or aggravating lung injury. Concurrently, the “gut-brain axis” theory posits that the gut microbiota can influence brain cognitive functions and behaviors through neural, immune and endocrine pathways.

The “triple-hit” hypothesis, which is based on these regulatory mechanisms, offers a novel perspective for understanding cognitive impairments caused by lung injury. The present study emphasizes the multifaceted role of the gut microbiota within the “gut-lung-brain axis” and demonstrates its capacity to impact cognitive functions through various mechanisms.

Recent studies have highlighted the role of specific metabolites in the pathogenesis of HPH. For instance, trimethylamine N-oxide (TMAO) has been demonstrated to intensify pulmonary hypertension (Huang et al., 2022). In the present study, 25 differential metabolites associated with HPH were identified, primarily involving the pyrimidine metabolism pathway. The administration of OFB treatment resulted in a substantial restoration of these metabolite levels, suggesting its potential as a metabolic regulator in HPH. Furthermore, correlation analysis revealed significant associations between gut microbiota and serum metabolites, thereby providing further evidence to support the hypothesis that gut microbiota dysbiosis contributes to metabolic alterations in HPH (Luo et al., 2023; Sanada et al., 2020).

It can be hypothesized that the therapeutic effects of OFB on HPH may also be related to its antioxidant and anti-inflammatory properties (Everard et al., 2013; Liao et al., 2023). As demonstrated in earlier research, OFB has been found to ameliorate hypoxia-induced lung injury by means of downregulating glucose-6-phosphate dehydrogenase (G6PD) and reducing oxidative stress (Xuefeng, 2025). In the present study, it was demonstrated that OFB treatment was able to restore redox homeostasis by means of regulating the levels of *N-acetylglucosamine*, a metabolite which has been identified as being involved in antioxidant defense (Ou et al., 2021). Furthermore, the administration of OFB treatment resulted in a reduction in the proliferation of endoplasmic reticulum and mitochondria in pulmonary artery smooth muscle cells, thereby indicating its protective effects against pulmonary artery remodeling.

In conclusion, this study provides valuable insights into the therapeutic potential of OFB in alleviating HPH by modulating gut microbiota and metabolites. It is recommended that future research endeavors focus on a more comprehensive exploration of the molecular mechanisms that underpin the therapeutic effects of OFB on HPH. For instance, the identification of a postprandial neuroimmune axis by Kim (2025), which links the gastrointestinal tract to type 2 immunity in the lung via brain-coordinated sensory and motor circuits, suggests a possible connection to the gut-lung axis. Further investigation is warranted to elucidate whether this gut-brain-lung axis plays a crucial role in the pathogenesis of HPH. Furthermore, the conduction of clinical trials is imperative to substantiate the therapeutic efficacy of OFB in human HPH patients, thereby facilitating its potential implementation in clinical settings.

## Conclusion

This study demonstrates that *Oxytropis falcata* Bunge can regulate gut microbiota dysbiosis and restore metabolic homeostasis in rats with hypoxic pulmonary hypertension. These findings highlight the potential therapeutic effects of OFB on HPH and provide a basis for further exploration of its underlying mechanisms.

## Data Availability

The datasets presented in this study can be found in online repositories and the Supplementary materials. The raw 16S rRNA sequencing data have been deposited into the NCBI Sequence Read Archive (SRA) under BioProject ID PRJNA1348596. The processed metabolomics dataset, which includes all peak intensities and compound identifications supporting the conclusions of this article, is available as Supplementary Material.
